# On the Relevance of Sophisticated Structural Annotations for Disulfide Connectivity Pattern Prediction

**DOI:** 10.1371/journal.pone.0056621

**Published:** 2013-02-15

**Authors:** Julien Becker, Francis Maes, Louis Wehenkel

**Affiliations:** 1 Bioinformatics and Modeling, GIGA-Research, University of Liege, Liege, Belgium; 2 Department of Electrical Engineering and Computer Science, Montefiore Institute, University of Liege, Liege, Belgium; 3 DTAI, Departement Computerwetenschappen, University of Leuven, Leuven, Belgium; Aberystwyth University, United Kingdom

## Abstract

Disulfide bridges strongly constrain the native structure of many proteins and predicting their formation is therefore a key sub-problem of protein structure and function inference. Most recently proposed approaches for this prediction problem adopt the following pipeline: first they enrich the primary sequence with structural annotations, second they apply a binary classifier to each candidate pair of cysteines to predict disulfide bonding probabilities and finally, they use a maximum weight graph matching algorithm to derive the predicted disulfide connectivity pattern of a protein. In this paper, we adopt this three step pipeline and propose an extensive study of the relevance of various structural annotations and feature encodings. In particular, we consider five kinds of structural annotations, among which three are novel in the context of disulfide bridge prediction. So as to be usable by machine learning algorithms, these annotations must be encoded into features. For this purpose, we propose four different feature encodings based on local windows and on different kinds of histograms. The combination of structural annotations with these possible encodings leads to a large number of possible feature functions. In order to identify a minimal subset of relevant feature functions among those, we propose an efficient and interpretable feature function selection scheme, designed so as to avoid any form of overfitting. We apply this scheme on top of three supervised learning algorithms: k-nearest neighbors, support vector machines and extremely randomized trees. Our results indicate that the use of only the PSSM (position-specific scoring matrix) together with the CSP (cysteine separation profile) are sufficient to construct a high performance disulfide pattern predictor and that extremely randomized trees reach a disulfide pattern prediction accuracy of 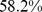 on the benchmark dataset SPX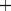, which corresponds to 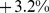 improvement over the state of the art. A web-application is available at http://m24.giga.ulg.ac.be:81/x3CysBridges.

## Introduction

A disulfide bridge is a covalent link resulting from an oxidation-reduction process of the thiol group of two cysteine residues. Both experimental studies in protein engineering [1]–[3] and theoretical studies [4], [5] showed that disulfide bridges play a key role in protein folding and in tertiary structure stabilization. The knowledge of the location of these bridges adds strong structural constraints to the protein, which enable to drastically reduce the conformational search space in the context of protein structure prediction. Due to the technical difficulties and the expensive cost of experimental procedures for determining protein structures (by x-ray crystallography, NMR or mass spectrometry), machine learning approaches have been developed to predict the formation of disulfide bridges in an automatic way.

**Figure 1:**
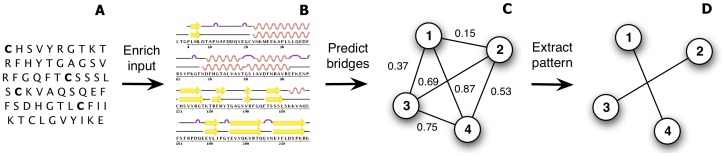
Three-step approach for disulfide pattern prediction. (A) an input primary structure, which contains four cysteine residues. (B) The sequence is first enriched using evolutionary information and sometimes structural-related predictions such as the secondary structure. (C) A bridge classifier, then, predicts disulfide bonding probabilities for each cysteine pair and finally (D) a graph matching algorithm extracts the disulfide pattern with maximal weight.

Given an input primary structure, the disulfide pattern prediction problem consists in predicting the set of disulfide bridges appearing in the tertiary structure of the corresponding protein. This problem can be formalized as an edge prediction problem in a graph whose nodes are cysteine residues, under the constraint that a given cysteine is linked to at most to a single other one. Most recent successful methods to solve this problem are pipelines composed of three steps which are illustrated in Figure 1. First, they enrich the primary structure using evolutionary information and sometimes structural-related predictions. Second, they apply a binary classifier to each pair of cysteines to estimate disulfide bonding probabilities. Finally, they use a maximum weight graph matching algorithm to extract a valid disulfide pattern maximizing the sum of these probabilities.

The central component of this three step pipeline is the binary classifier that predicts bonding probabilities for all cysteine pairs. The wide majority of available binary classification algorithms cannot process complex objects such as cysteine pairs natively, hence they require the user to encode such objects into vectors of (categorical or numerical) features. Since the way to perform this encoding typically has a major impact on the classification accuracy, a large body of work has been devoted to studying different feature representations for cysteines and cysteine-pairs. However, it is often the case that these different studies rely on different kinds of binary classifiers and slightly differ in their experimental protocol. Therefore, the comparison of the conclusions of these works is difficult. In consequence, the relevance of some features is still a subject under heavy debate. It is for example not clear whether the use of (predicted) secondary structure or (predicted) solvent accessibility can significantly improve disulfide pattern predictors [6]–[8].

The main contribution of this paper is an extensive study which aims at establishing the relevance of various structural-related annotations and of various feature encodings in the context of a disulfide pattern predictor such as the one presented in Figure 1. We consider various structural annotations, some which were already studied in the context of disulfide pattern prediction – position-specific scoring matrix, secondary structure and solvent accessibility – and some others which are more original in this context: 8-class secondary structure, disordered regions and structural alphabet. For each such annotation, we consider four different procedures in order to encode it as a feature vector. The combination of annotations with feature encodings leads to a large set of possible feature functions. In order to identify a minimal subset of feature functions that are relevant to disulfide pattern prediction, we introduce a tractable and interpretable feature selection methodology, based on forward selection of feature functions. We adopt a computational protocol that avoids any risk of overfitting and apply our approach in combination with two usual classifiers: k-nearest neighbors (kNN) and support vector machines (SVM), as well as with one classifier, which was not yet considered for disulfide pattern prediction: extremely randomized trees (ET) [9].

As a result of this study, we show that only a very limited number of feature functions are sufficient to construct a high performance disulfide pattern predictor and that, when using these features, extremely randomized trees reach a disulfide pattern accuracy of 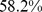 on the benchmark dataset SPX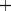, which corresponds to 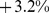 improvement over the state of the art. However, since SPX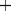 only contains proteins with at least one intrachain disulfide bridge, we further consider the more heterogeneous and less redundant benchmark dataset SPX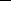 which also contains a significant number of proteins without any intrachain bridge. We then investigate the behavior of our disulfide pattern predictor on both datasets by coupling it with filters predicting the presence of intrachain bridges and the bonding states of individual cysteines. We consider both the case where bonding states are known a priori and the case where bonding states are estimated thanks to another predictor. We show that predicting the bonding states significantly improves our disulfide pattern predictor on SPX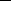, but slightly degrades it on SPX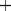. When the bonding states are known a priori, we reach very high accuracies: 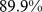 on SPX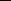 and 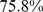 on SPX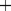.

The following two sections give an overall view of related work by first discussing multiple sub-problems of disulfide pattern prediction and then presenting the kinds of features that have been proposed to describe cysteines and cysteine pairs in supervised learning approaches. We refer the reader to [10] for an extensive recent overview of the field.

### Disulfide bridge related prediction problems

While the ultimate goal of disulfide bridge prediction is to infer correctly the whole connectivity pattern of any protein from its primary sequence, several researchers have focused on intermediate simpler sub-problems, which are detailed below.

#### Chain classification

This sub-problem aims at predicting for a given protein, whether (a) none of its cysteines participate to a disulfide bridge, (b) some of its cysteines are involved in disulfide bridges or (c) all of its cysteines are involved in disulfide bridges. Frasconi *et al.* [11] proposed a support vector machine classifier to solve this task. Fiser *et al.* [12] have exploited the key fact that free cysteines (not involved in any bond) and oxidized cysteines (involved in a bond but not necessarily an intra-chain disulfide bridge) rarely co-occur and shown that theirs sequential environments are different. From those observations, subsequent studies have reduced this sub-problem to a binary classification task: (a) or (c).

#### Cysteine bonding state prediction

This second commonly studied sub-problem consists in classifying cysteines into those that are involved in a disulfide bridge and those that are not. To solve this binary classification problem, several machine-learning algorithms were proposed such as multi-layer neural networks [13], two-stage support vector machines that exploit chain classification predictions [11] and hidden neural networks [14].

#### Disulfide bonding prediction

While chain classification works at the protein level and cysteine bonding state prediction works at the cysteine level, disulfide bonding prediction works at the level of cysteine pairs and aims at predicting the probability that a specific pair of cysteines will form a disulfide bridge during protein folding. Depending on the studies, some authors assume to have an *a priori* knowledge on the bonding state of isolated cysteines. This prior knowledge can be the actual state [15]–[17] or a prediction made by a cysteine bonding state predictor [18].

#### Disulfide pattern prediction

Once one or several of the previous tasks have been solved, the most challenging step is to predict the disulfide connectivity pattern. Fariselli *et al.* [19] were the first to relate the problem of predicting the disulfide pattern to a maximal weight graph matching problem. Several authors have since adopted this approach and proposed disulfide pattern predictors that fit into the three step pipeline of Figure 1. Baldi *et al.* [6], [20] have used two-dimensional recursive neural networks to predict bonding probabilities, which are exploited by a weighted graph matching algorithm. Lin *et al.* [7], [21] used the same graph matching approach while predicting bonding probabilities with support vector machines.

### Features for cysteines and cysteine pairs

Machine learning algorithms are rarely able to process complex objects such as cysteine pairs directly, hence it is necessary to define a mapping from these objects to vectors of features. A large body of research on disulfide bridge prediction has been devoted to the analysis of such encodings into feature vectors.

In 2004, Vullo *et al.* [15] suggested to incorporate evolutionary information into features describing cysteines. For each primary sequence, they generate a position-specific scoring matrix (PSSM) from a multiple alignment against a huge non-redundant database of amino-acid sequences. This evolutionary information was shown to significantly improve the quality of the predicted disulfide bridges, which led the large majority of authors to use it in their subsequent studies. Generally, the PSI-BLAST program [22] is used to perform multiple alignments against the SWISS-PROT non-redundant database [23].

Zhao *et al.* [24] introduced cysteine separation profiles (CSPs) of proteins. Based on the assumption that similar disulfide bonding patterns lead to similar protein structures regardless of sequence identity, CSPs encode sequence separation distances among bonded cysteine residues. The CSP of a test protein is then compared with all CSPs of a reference dataset and the prediction is performed by returning the pattern of the protein with highest CSP similarity. This approach assumes to have an *a priori* knowledge on the bonding state of cysteines. In this paper, we introduce a slightly different definition of CSPs based on separation distances among all cysteine residues (see *Candidate feature functions*).

From the earlier observation that there is a bias in the secondary structure preference of bonded cysteines and non-bonded cysteines, Ferrè *et al.* [8] have developed a neural network using predicted secondary structure in addition to evolutionary information. Cheng *et al.* [6] proposed to also include predictions about the solvent accessibility of residues. The predictions of secondary structure and/or solvent accessibility used in their experiments were however not accurate enough to obtain significant performance improvements. Nevertheless, they observed that using the *true values* of secondary structure and solvent accessibility can lead to a small improvement of 1%. More recently, Lin *et al.* [7] proposed an approach based on support vector machines with radial basis kernels combined with an advanced feature selection strategy. They observed a weak positive influence by using predicted secondary structure descriptors, but their experimental methodology could suffer from overfitting so that this result should be taken with a grain of salt. Indeed, in this study, the same data is used both for selecting features and for evaluating the prediction pipeline. As detailed in [25], proceeding in this way often lead to an overfitting effect and hence to over-optimistic scores. Notice that the three studies [6]–[8] were all based on the secondary structure predicted by the PSIPRED predictor [26].

More recently, Savojardo *et al.* [27] reported an improvement of their predictive performance by taking into consideration the relevance of protein subcellular localization since the formation of disulfide bonds depends on the ambient redox potential.

## Materials and Methods

### Notations and problem statement

This section introduces notations and formalizes the disulfide pattern prediction problem. Let 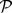 be the space of all proteins described by their primary structure and 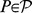 one particular protein. We denote 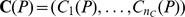 the sequence of 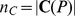 cysteine residues belonging to protein 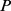, arranged in the same order as they appear in the primary sequence. A disulfide bonding connectivity pattern (or disulfide pattern) is an undirected graph 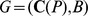 whose nodes 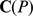 are cysteines and whose edges 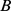 are the pairs of cysteines 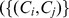 that form a disulfide bridge.

**Figure 2:**
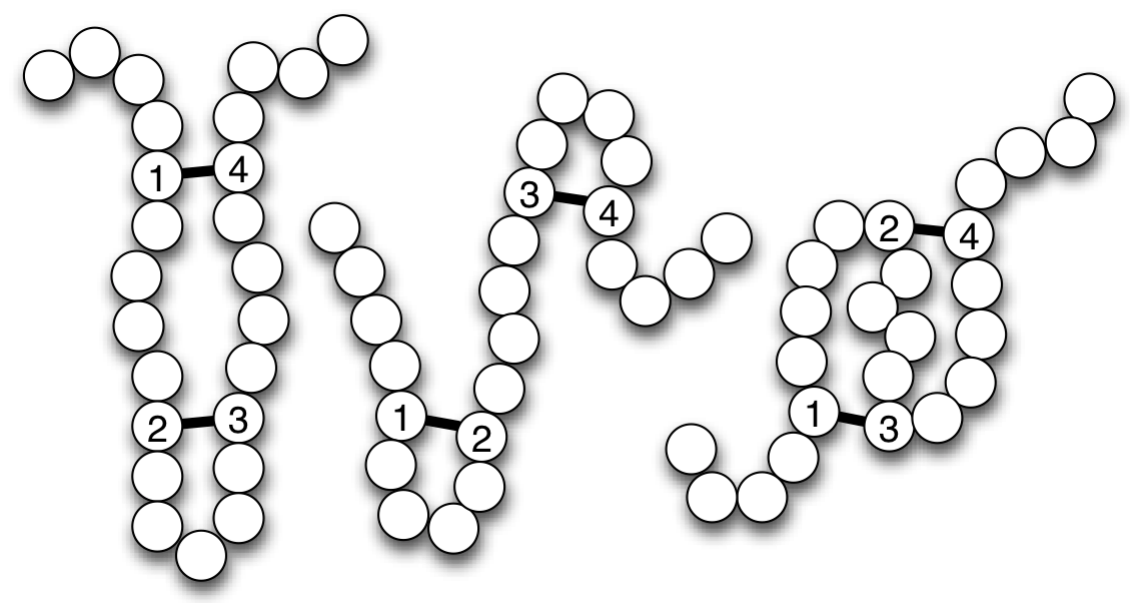
Example of disulfide patterns. A protein with two disulfide bridges and its three possible disulfide connectivity patterns.

Since a given cysteine can physically be bonded to at most one other cysteine, valid disulfide patterns are those that respect the constraint 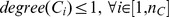. This constraint enables to trivially derive an upper bound on the number 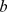 of disulfide bridges given the number of cysteines: 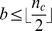, where 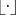 is the floor function. If we know in advance the number 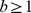 of disulfide bridges, we can derive the number of valid disulfide patterns using the following closed form formula [28]:
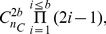
(1)where 
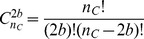
 denotes the number of possible subsets of size 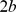 of the set of 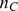 cysteines. As an example, a protein with 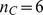 cysteines and 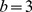 bridges has 15 possible disulfide patterns and a protein with 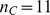 cysteines and 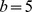 bridges has 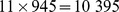 possible patterns. Figure 2 illustrates the three possible disulfide connectivity patterns of a protein with four cysteines and two disulfide bridges.

When the number of bridges is unknown, the number of possible disulfide connectivity patterns for a protein 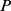 with 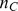 cysteines becomes
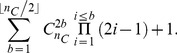
(2)Note that the term 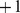 represents the case where no cysteine residue is bonded. As an example, a protein with 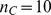 cysteines has 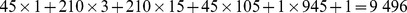 possible valid disulfide patterns.

We adopt a supervised-learning formulation of the problem, where we assume to have access to a dataset of proteins (represented by their primary structure) with associated disulfide patterns. We denote this dataset 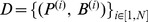, where 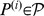 is the 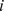-th protein and 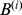 is the set of disulfide bridges associated to that protein. We also denote 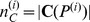 the number of cysteines belonging to the protein 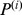. Given the dataset 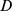, the aim is to learn a disulfide pattern predictor 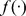: a function that maps proteins 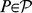 to sets of predicted bridges 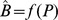. Given such a predicted set, we can define the predicted connectivity pattern as following: 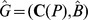.

We consider two performance measures to evaluate the quality of predicted disulfide patterns: 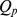 and 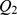. 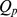 is a protein-level performance measure that corresponds to the proportion of entirely correctly predicted patterns:
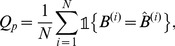
(3)where 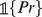 is the indicator function whose value is 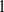 if 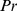 is true or 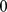 otherwise. 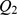 is a cysteine-pair level performance measure that corresponds to the proportion of cysteine pairs that were correctly labeled as *bonded* or *non-bonded*:
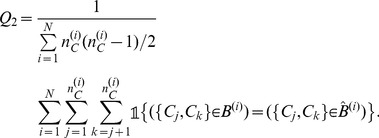
(4)


Note that both 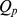 and 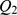 belong to the interval 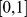 and are equal to 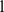 in case of perfectly predicted disulfide patterns. While the ultimate goal of disulfide pattern prediction is to maximize 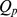, we will also often refer to 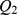 since, in the pipeline depicted in Figure 1, 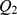 is directly related to the quality of the cysteine pair classifier.

### Disulfide pattern prediction pipeline

This section first presents the datasets and the five kinds of structural-related predictions we consider. It then details the different steps of our prediction pipeline: the dataset annotation, the pre-processing step that enriches the primary structure with evolutionary information and structural-related annotations, the classification step of cysteine pairs that predicts bridge bonding probabilities and the post-processing step that constructs a disulfide pattern from these probabilities using maximum weight graph matching.

#### Dataset and annotations

In order to assess our methods, we use two datasets that have been built by Cheng *et al.* [6] and extracted from the Protein Data Bank [29]. The first one, SPX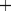, is a collection of 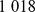 proteins that contain at least 12 amino acids and at least one intrachain disulfide bridge. We use this dataset for the problem of pattern prediction. However, since it does not contain any protein without disulfide bridges it is not adapted to address chain classification and cysteine bonding state prediction. For these tasks, we use the other dataset, SPX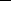, which is made of 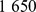 proteins that contain no disulfide bridge and 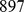 proteins that contain at least one bridge. In order to reduce the over-representation of particular protein families, both datasets were filtered by UniqueProt [30], a protein redundancy reduction tool based on the HSSP distance [31]. In SPX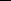, Cheng *et al.* used a HSSP cut-off distance of 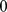 for proteins without disulfide bridge and a cut-off distance of 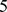 for proteins with disulfide bridges. In SPX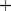, the cut-off distance was set to 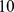. To properly compare our experiments with those of Cheng *et al.*, we use the same train/test splits as they used in their paper. Statistics of the two datasets are given in Table 1.

**Table 1:** Dataset statistics.

	Proteins				Cysteines			Bonds per protein
	All	None	Mix	Total	Positive	Negative	Total	
**SPX** 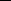	757	1 650	140	2 547	4 942	7 844	12 786	0.97 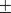 1.78
**SPX** 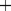	718	0	300	1 018	5 082	901	5 983	2.50 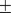 2.14

*All*: proteins in which all cysteines are bonded. *None*: proteins with no disulfide bridges. *Mix*: proteins with both bonded cysteines and non-bonded cysteines. *Positive*: number of bonded cysteines. *Negative*: number of non-bonded cysteines.

We enrich the primary structure (denoted as 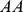) by using two kinds of annotations: evolutionary information in the form of a position-specific scoring matrix (PSSM) and structural-related predictions, such as predicted secondary structure or predicted solvent accessibility. We computed the PSSMs by running three iterations of the PSI-BLAST program [22] on the non-redundant NCBI database. To produce structural-related predictions, we use the iterative multi-task sequence labeling method developed by Maes *et al.* [32]. This method enables to predict any number of structural-related properties in a unified and joint way, which was shown to raise state of the art results. We consider here five kinds of predicted annotations: secondary structure (SS3, 3 labels), DSSP secondary structure (SS8, 8 labels), solvent accessibility (SA, 2 labels), disordered regions (DR, 2 labels) and a structural alphabet (StAl, 27 labels, see [33]). The two versions of secondary structure give two different levels of granularity. The structural alphabet is a discretization of the protein backbone conformation as a series of overlapping fragments of four residues length. This representation, as a prediction problem, is not common in the literature. Here, it is used as a third level of granularity for local 3D structures. To our best knowledge, predicted DSSP secondary structure, predicted disordered regions and structural alphabet annotations have never been investigated in the context of disulfide pattern prediction.

In order to train the system of Maes *et al.*, we rely on supervision information computed as follows: secondary structures and solvent accessibility are computed using the DSSP program [34], disordered regions and structural alphabet are computed by directly processing the protein tertiary structure. Since the disorder classes are not uniquely defined, we use the definition of the CASP competition [35]: segments longer than three residues but lacking atomic coordinates in the crystal structure were labelled as *disordered* whereas all other residues were labelled as *ordered*.

Note that it is often the case that supervised learning algorithms behave differently on training data than on testing data. For example, the 1-nearest neighbor algorithm always has a training accuracy of 100%, while its testing accuracy may be arbitrarily low. In order to assess the relevance of predicted annotations, we expect our input enrichment step to provide “true” predictions, *i.e.*, representative of predictions corresponding to examples that were not part of training data.

We therefore use the cross-validation methodology proposed in [36] that works as follows. First, we randomly split the dataset into ten folds. Then, in order to generate “true” predictions for one fold, we train the system of Maes et al. on all data except this fold. This procedure is repeated for all ten folds and all predictions are concatenated so as to cover to whole dataset.

Table 2 reports the cross-validation accuracies that we obtained with this procedure. The default scoring measure is label accuracy, *i.e.*, the percentage of correctly predicted labels on the test set. Since disordered regions labeling is a strongly unbalanced problem, label accuracy is not appropriate for this task. Instead, we used a classical evaluation measure for disordered regions prediction: the Matthews correlation coefficient [37].

**Table 2:** Cross-validated accuracies of annotations.

Annotation	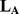	Measure	SPX 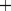	SPX 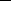
Secondary structure	3	Accuracy	73.50% 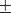 0.68%	68.00% 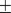 2.61%
Secondary structure	8	Accuracy	55.60% 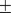 0.76%	57.83% 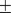 2.10%
Solvent accessibility	2	Accuracy	77.45% 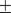 0.54%	77.82% 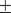 0.30%
Disorder regions	2	MCC	0.892 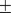 0.03	0.352 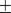 0.05
Structural alphabet	27	Accuracy	19.01% 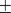 0.30%	21.32% 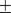 0.47%

The scoring measure is label accuracy, *i.e.*, the percentage of correctly predicted labels on the test set except for disordered regions that use the Matthews correlation coefficient (MCC).

#### Candidate feature functions

The feature generation step aims at describing cysteine pairs in an appropriate form for classification algorithms. This encoding is performed through *cysteine-pair feature functions* 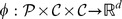 that, given a protein 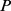 and two of its cysteines 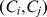, computes a vector of 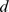 real-valued features. In our experiments, we extracted cysteine-pairs 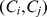 in such a way that 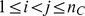, where 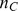 is the number of cysteine residues of 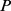. Consequently, we extract 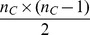 cysteine-pairs from 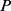. The purpose of the feature selection methodology described in the next section is to identify a subset of relevant 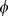 functions among a large panel of candidate ones that we describe now.

Our set of candidate feature functions is composed of primary-structure related functions and annotation related functions. The former are directly computed from the primary structure alone and are the following ones:

- *Number of residues*: computes one feature which is the number of residues in the primary structure.
- *Number of cysteines*: computes one feature which is the number of cysteine residues in the primary structure.
- *Parity of the number of cysteines*: computes one feature which indicates whether the number of cysteines is odd or even.
- *Relative position of cysteines*: computes two features which are the residue indices of cysteines 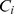 and 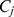, denoted 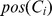 and 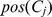, divided by the protein length.
- *Normalized position difference*: returns one feature which corresponds to the number of residues separating 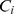 from 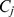 in the primary structure, *i.e.*, 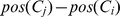, divided by the protein length. Note that as 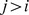 and therefore 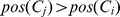, this difference is always greater than zero.
- *Relative indices of cysteines*: computes two features which are the cysteine indices 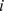 and 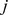 divided by the number of cysteines.
- *Normalized index difference*: computes one feature which corresponds to the number of cysteines separating 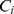 from 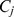 divided by the number of cysteines.
- *Cysteine separation profile window*: computes one feature per cysteine 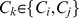 and per relative position 
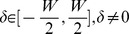
 whose value is the position difference 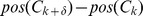 divided by the protein length, where 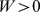 is called the window size parameter.

Annotation-related feature functions are defined for each type of annotation 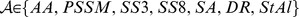 of the residues of the protein 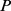. We denote by 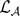 the set of labels corresponding to annotation 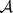 and by 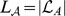 the size of this set. For our annotations, we have: 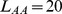, 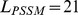 (the twenty amino acids and the gap), 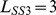, 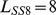, 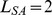, 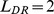 and 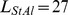. For a given primary structure of length 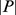, an annotation 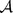 is represented as a set of probabilities 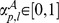 where 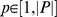 denotes the residue index and 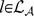 is a label.

Note that in the general case, 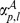 probabilities may take any value in range 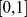 to reflect uncertainty about predictions. Since the primary structure (AA) is always known perfectly, we have:
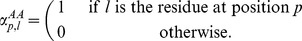



For each annotation 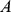, we have four different feature functions:

- *Labels global histogram*: returns one feature per label 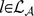, equal to 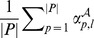.
- *Labels interval histogram*: returns one feature per label 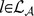 equal to 
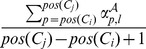
.
- *Labels local histogram*: returns one feature per label 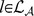 and per cysteine 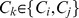, equal to 
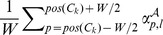
 and one special feature equal to the percentage of out-of-bounds positions, *i.e.*, positions 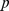 such that 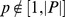.
- *Labels local window*: returns one feature per label 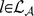, per cysteine 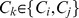 and per relative position 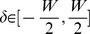, equal to 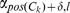. When the position is out-of-bounds, *i.e.*, 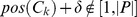, the feature is set to 0.

**Figure 3:**
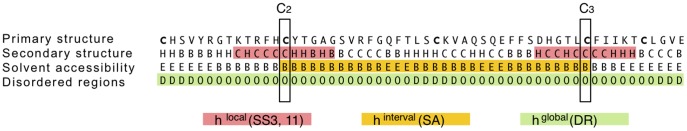
Example of local, interval and global histograms. 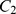
 and 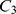 are the two cysteines of interest. In red, we show the labels local histograms of size 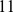 of the secondary structure 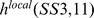. In yellow, we show the labels interval histogram of the solvent accessibility annotation 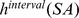. In green, we show the global histogram of the disordered regions sequence 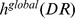.

Our candidate feature functions are summarized in Table 3. Note that three of them are parameterized by window size parameters. Figure 3 shows an illustration of the three kinds of histograms. We will see how to tune window sizes and how to select a minimal subset of feature functions in the next section.

**Table 3:** Feature functions used in our experiments to encode cysteine pairs.

Symbol	Parameter	d	Description
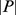	-	1	Number of residues
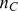	-	1	Number of cysteines
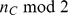	-	1	Parity of the number of cysteines
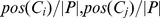	-	2	Relative position of cysteines
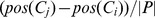	-	1	Position difference
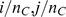	-	2	Indices of cysteines
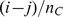	-	1	Index difference
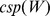	window size	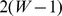	Cysteine separation profile window
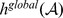	-	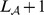	Labels global histogram
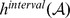	-	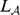	Labels interval histogram
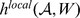	window size	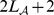	Labels local histogram
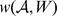	window size	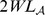	Labels local window

Symbols, parameters, number of features (d) and description of our candidate feature functions. Top: feature functions that are directly computed from the primary structure. Bottom: feature functions defined for every kind of annotation 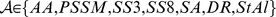.

#### Cysteine pair classifiers

Let 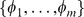 be a subset of the candidate feature functions described above and let 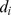 denote the dimensionality of the 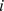-th function of this set. A cysteine pair classifier processes feature vectors of dimension 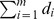, in order to predict disulfide bonding probabilities. In this study, we consider three such binary classifiers:

- *K-nearest neighbors* (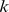
NN) is a simple and well-known method for classification. In order to determine the disulfide bonding probability of a new example, the algorithm first search for the 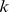 nearest training samples and then returns the frequency of bonded cysteines among these neighbors. The distance between two feature vectors 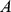 and 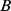 is computed using a normalized version of the l2-norm, which is defined as follows:
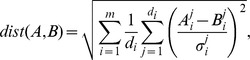
where 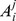 and 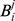 denote the 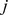-th components of the 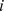-th feature generator 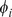, and where 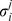 denotes the empirical standard deviation of this component, computed on the training data. Since we are concataining feature functions with very different dimensionalities (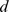 varies from 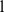 to 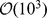), the effect of the traditional l2-norm would be largely dominated by high-dimensional feature functions. The term 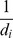 enables to avoid this problem. Dividing by the standard deviations 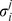 is a classical strategy to be less dependent on the domain of the different features.
- *Support vector machines* (SVM) is also a well-established method that constructs a hyperplane that maximizes the distance to the nearest training samples of any class in a high-dimensional space. The method has one hyper parameter that is a regularization constant 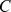. Among the common functions used to cope with non-linear feature interactions, called *kernel functions*, we use the Gaussian radial basis function 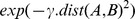, where 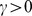 is a bandwidth hyper-parameter and where 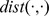 is the same norm as previously. Note that previous studies on disulfide pattern prediction [7], [38] also relied on the Gaussian radial basis function. In our experiments, we used the well-known LibSVM implementation [39]. In order to convert SVM predictions into probabilities, we use the default probability estimation method of LibSVM, which was proposed by Platt [40] and Wu *et al.* [41].
- *Extremely randomized trees* (ETs). This tree-based ensemble method, proposed by Geurts *et al.* [9], is similar to the popular Random Forests approach [42]. The main differences with the latter are that ETs does not rely on bootstrap replicates (unlike the Random Forests method, each tree is built using all learning samples), and that cut-points are selected in a random fashion, which was shown to lead to better generalization performances. The method has three hyper-parameters: 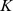, the number of random splits tested per node creation, 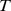, the number of trees composing the ensemble, and 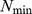, the minimum number of samples required to allow for splitting a node. We use the probabilistic version of ETs, in which each leaf is associated to a bonding probability, which is the empirical proportion of bonded cysteine pairs among the training samples associated to that leaf. In order to make one prediction, we traverse each of the 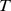 trees and return the average of the bonding probabilities associated to the corresponding 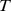 leaves. To our best knowledge, tree-based ensemble methods, and in particular ETs, were not yet applied to disulfide connectivity pattern prediction, despite the fact that several studies have shown that these methods very often outperform other methods such as support vector machines or neural network [43].

#### Maximum weight graph matching

Given bonding probabilities for every cysteine pair of a protein, the aim of this last step of the disulfide pattern prediction pipeline is to select a subset of disulfide bridges so as to respect the constraint 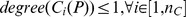. As proposed previously, this problem is formalized as a maximum weight graph matching problem: the weight of a disulfide pattern is defined as the sum of probabilities attached to its edges and the aim is to find the valid pattern with maximal weight.

A naive solution to solve the maximum weight graph matching problem is to perform an exhaustive search over all valid disulfide patterns. The complexity of this procedure is however exponential in the number of cysteines, which is problematic for large proteins. This issue is often solved using the maximum weight matching algorithm of Gabow [44] whose time complexity is cubic w.r.t. the number of cysteines 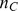 and whose space complexity is linear w.r.t. 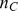. In our experiments, we used Blossom V, which is a more recent and optimized implementation proposed by Kolmogorov [45].

Notice that, because this algorithm searches for a full matching, *i.e.*, where each cysteine is associated to another one, it cannot be directly applied on proteins that have an odd number 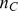 of cysteines. To deal with such proteins, we run the matching algorithm on each one of the 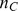 subsets of 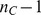 cysteines and select the solution with maximal weight.

### Forward feature function selection

This section describes our forward feature function selection algorithm, which aims at determining a subset of relevant feature functions among those described above. Feature selection is an old topic in machine learning and a common tool in bioinformatics [46]. Our feature selection problem departs from traditional feature selection w.r.t. three unique aspects:

- *Feature function selection*: we want to select feature functions rather than individual features. Given that feature functions can be parameterized by window sizes, our algorithm has to perform two tasks simultaneously: determining a subset of feature functions and determining the best setting for associated window sizes.
- *Insertion in a pipeline*: we want to optimize the performance 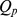 of the whole pipeline rather than the accuracy 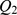 of the classifier for which we perform feature selection. Preliminary studies have shown that these two performance measures are not perfectly correlated: a binary classifier with higher accuracy can lead to worse disulfide pattern predictions when combined with the graph matching algorithm, and conversely.
- *Interpretability*: our approach not only aims at constructing a pipeline maximizing 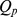, but also at drawing more general scientific conclusions on the relevance of various annotations of the primary structure. We thus require the result of the feature selection process to be interpretable.

In order to fulfill these requirements, we adopt a *wrapper* approach that repeatedly evaluates feature function subsets by cross-validating the whole pipeline and that is directly driven by the cross-validated 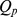 scores. In order to obtain interpretable results, we rely on a rather simple scheme, which consists in constructing the feature function set greedily in a forward way: starting from an empty set and adding one element to this set at each iteration.

In order to treat feature functions with parameters and those without parameters in an unified way, we express the feature functions as a set of parameterized feature functions 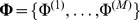 where each 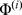 contains a set of alternative feature functions 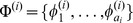. In the case where the feature function has no parameters (*e.g.*, number of residues or labels global histogram), this set is a singleton 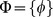. Otherwise, when the feature function is parameterized by a window size, there is one alternative per possible window size, *e.g.*, 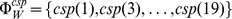.

Our forward feature function selection approach is depicted in Algorithm. We denote by 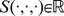 the objective function that evaluates the 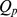 score associated to a given set of feature functions, based on a cysteine pair classifier 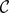 and a dataset of proteins 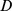. In our experiments, this objective function is computed by performing a 10-fold cross-validation of the whole prediction pipeline and by returning the test 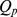 scores averaged over the ten folds.

#### Algrithm 1

Forward feature function selection algorithm.

*Given* a set of parameterized feature functions 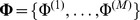


*Given* an objective function 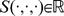


*Given* a cysteine pair classifier 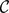


*Given* a dataset 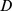


1: 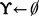 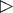
initial empty feature function set

2: **repeat**

3: 
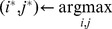
 
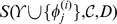
 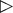
evaluate candidate 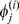 functions

4: 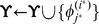 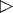
add the best feature function

5: 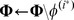 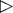
remove the best parameterized feature function

6: **until** some stopping criterion is fulfilled

7:**return** 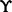 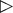
return feature function set

The feature function is first initialized to an empty set 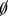 (line 1). Each iteration then consists in inserting a candidate feature functions 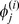 taken in the set 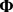 into 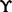. For this, we try to add each candidate 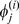 to the current feature function set and select the best feature function w.r.t. the obtained cross-validation 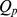 scores (line 3). This feature function is then inserted into 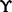 (line 4) and the corresponding set of alternatives 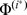 is removed from 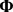. After a given stopping criterion is fulfilled, the constructed function set 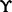 is returned (line 7). In our experiments, this stopping criterion is simply a fixed number of iterations. An alternative consists in stopping the algorithm when no additional feature functions enable to improve the 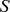 score.

Note that due to its greedy nature, our feature selection may fall into local minima. However, compared to traditional feature selection, it may be the case that selecting feature functions instead of individual features significantly reduces the importance of this problem (the dimensionality of our search problem is much smaller than in the case of individual feature selection). We show in the next section that this algorithm is a tractable feature function selection approach that provides interpretable results, from which we can draw some general conclusions about the relevance of primary structure annotations.

## Results: Disulfide Pattern Prediction

This section describes our experimental study on disulfide pattern prediction using the SPX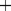 benchmark dataset. We first make an overall comparison of the three binary classification algorithms described previously and show that extremely randomized trees lead to significantly better results than the two other algorithms. We then apply our forward feature function selection approach using this algorithm and show that only a few feature functions are sufficient to construct a high performance disulfide pattern predictor. We finally compare this predictor with the state of the art and propose an analysis of the sensitivity of extremely randomized trees w.r.t. their hyper-parameters. Note that, for the moment, our prediction pipeline always tries to construct fully connected disulfide patterns and that it does not enable predicting partially connected disulfide patterns. We address this issue in the next section, by coupling our predictor with filters based on the bonding state of individual cysteines.

### Comparison of the cysteine pair classifiers

Comparing cysteine pair classifiers in our context is not trivial for two reasons. First, we are primarily interested in the 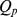 score of the whole prediction pipeline rather than in the classification accuracy. Second, we do not have a fixed feature representation and different classification algorithms may require different feature function sets to work optimally. To circumvent these difficulties, we compare cross-validated 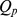 scores obtained with the three classifiers on a large number of randomly sampled feature function sets. To sample a feature function set of size 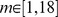, we proceed as follows. First, we draw a subset 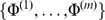 from 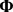. Then, for each member 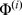 of this subset, we select a feature function 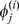, using the following rules: (i) local window sizes are sampled according to the Gaussian distribution 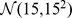, (ii) local histogram sizes are sampled according to 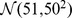 and (iii) CSP window sizes are sampled from 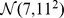. These values were chosen according to preliminary studies using the three classifiers.

We set the hyper-parameters in the following way:

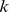
 *NN*. By studying the effect of 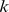, we found out that large values of 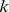 drastically decrease the performance of kNN and low values do not enable to distinguish patterns well since the set of possible predicted probabilities is limited to 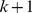 values. In the following, we use the default value 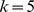, which we found to be a generally good compromise.
- *SVM*. It turns out that the best setting for 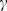 and 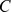 is highly dependent on the chosen feature function set. For each tested set of feature functions, we thus tuned these two parameters by testing all combinations of 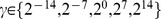 and 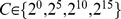 and by selecting the values of 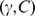 that led to the best 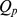 scores.
- *ETs*. We use a default setting that corresponds to an ensemble of 1 000 fully developed trees (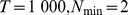) and 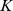 is set to the square root of the total number of features 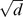, as proposed by Geurts *et al* [9].

**Figure 4:**
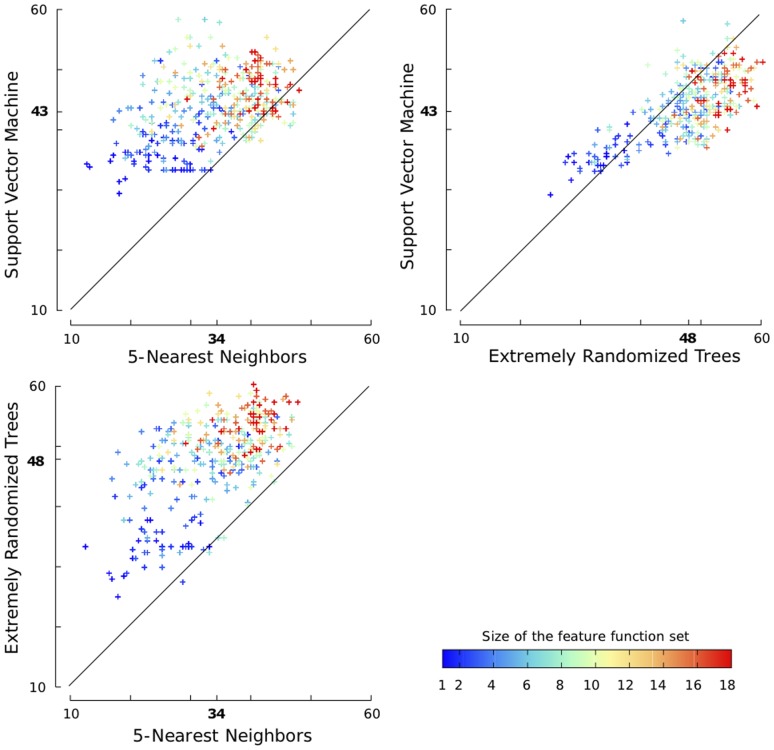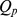 scores for three binary classification algorithms and randomly sampled feature function sets. The experiments are performed on the SPX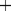 dataset. In bold, the means of the classifiers. The diagonal lines indicate situations where both classifiers have the same score. The experiments are color-encoded w.r.t. the size of their feature function set.

The results of our comparison on SPX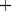 are given in Figure 4. As a first remark, note the large range in which the 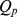 scores lie: from 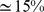 to 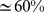. This shows that all three classifiers are highly sensitive to the choice of the features used to describe cysteine pairs, which is a major motivation for our work on feature function selection. The experiments are color-encoded w.r.t the size 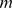 of their feature function set. This color-encoding enables us to notice that, in general, larger feature function sets lead to better classifiers.

The mean and standard deviations of these results are 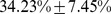 for kNN classifiers, 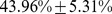 for SVM classifiers and 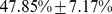 for ETs classifiers. In 73.25% of the experiments, the best pattern accuracy is given by ETs and in 20.35% of them by SVMs. In the remaining 6.40% experiments, exactly the same number of disulfide patterns were correctly predicted by ETs and SVM. 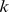
NN was always outperformed by the other two classifiers. We have used the paired 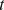-test to assess the significance of the out-performance of ETs. The 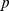-value against 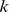
NN is 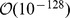 and the 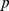-value against SVM is 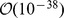, which make it clear that ETs significantly outperform 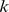
NN and SVM. Moreover, ETs work well with a default setting contrarily to SVM that required advanced, highly time-consuming, hyper-parameters tuning.

Given those observations, we proceed in the remainder of this study by restricting to the ETs method.

### Feature functions selection

We now apply our feature function selection approach on top of extremely randomized trees. We rely on the set of parameterized feature functions 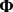 described in Table 3 and consider the following window size values:

- *Cysteine separation profile window*: 1, 3, 5, 7, 9, 11, 13, 15, 17, 19.
- *Local histograms*: 10, 20, 30, 40, 50, 60, 70, 80, 90.
- *Local windows*: 1, 5, 9, 11, 15, 19, 21, 25.

This setting leads to a total of 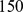 candidate features functions. As cysteine pair classifier, we use ETs with the same default setting as previously (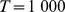, 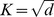, 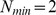).

The simplest way to apply our algorithm would be to apply it once on the whole SPX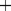 dataset. By proceeding in this way, the same data would be used for both selecting the set of feature functions and assessing the quality of this selected set. It has been shown that this approach is biased due to using the same data for selecting and for evaluating and that it could lead to highly over-estimated performance scores [25].

To avoid this risk of overfitting, we adopted a more evolved approach, which consists in running the feature selection algorithm once for each of our 10 different train/test splits. In this setting, the whole feature selection algorithm is executed on a training dataset composed of 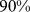 of the data and the generalization performance of the selected feature functions is evaluated using the remainder 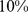 of data. There are thus two different objective functions. We call *cross-validated score* the value returned by 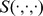, *i.e.*, the 10 cross-validated 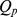 score using 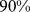 of the data, and we call *verification score* the 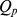 score computed over the remainder 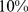 of the data.

**Figure 5:**
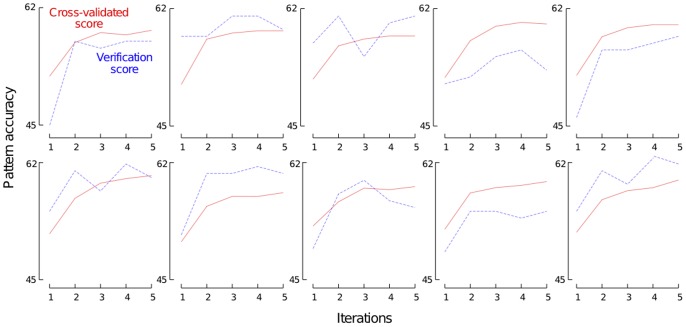
Forward feature function selection with 10 train/test splits of the SPX 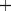 dataset. Each figure reports the results of five iterations of our forward feature function selection algorithm on one of ten train/test splits. Solid red lines are the cross-validated scores and dashed blue lines are the verification scores.

Figure 5 shows the evolution of the cross-validated score and the verification score for five iterations of the feature selection algorithm on each of the 10 train/test splits. Note that, since the cross-validated score is the score being optimized, its value increases at every iteration of each of the 10 runs. The evolution of the verification score, which represents the true generalization performance, is far from being so clear, as, in most cases, the optimum is not located after the fifth iteration.

Table 4 reports the selected feature functions for each of the 10 runs. We observe that the first selected feature function is always 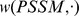 with a window size varying in 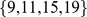. This means that, taken alone, the best individual feature function is always a window over the position-specific scoring matrix. The fact that this results was observed during each run is very strong, since the selection algorithm has to select between 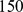 different functions. Similarly, the second selected feature function is always 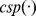 with a window size varying in 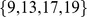.

**Table 4:** Forward feature functions selection with 10 train/test splits of the SPX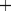 dataset.

Fold	Iteration 1	Iteration 2	Iteration 3	Iteration 4	Iteration 5
1	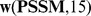	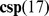	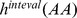	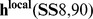	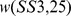
2	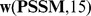	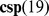	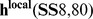	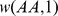	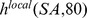
3	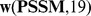	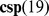	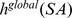	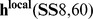	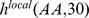
4	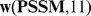	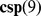	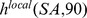	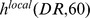	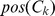
5	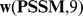	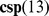	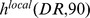	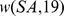	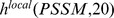
6	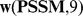	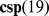	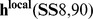	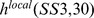	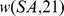
7	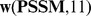	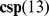	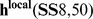	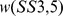	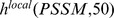
8	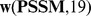	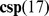	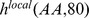	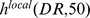	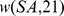
9	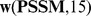	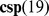	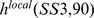	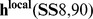	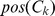
10	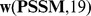	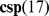	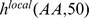	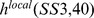	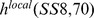
Mean					
Cross-validated	51.8% 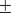 0.64%	56.9% 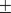 0.63%	58.3% 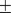 0.67%	58.6% 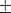 0.84%	58.9% 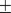 0.75%
Verification	51.6% 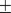 4.19%	57.8% 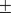 2.83%	57.4% 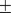 2.22%	58.7% 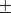 2.83%	58.0% 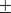 2.72%

In bold, the most frequent feature function (without consideration of the window size parameters) of each iteration. *Mean*: averages over the ten cross-validated scores and the ten verification scores.

After the two first iterations, the selected feature functions become more disparate and only lead to tiny improvements. This probably indicates that the system starts to overfit, by selecting feature functions that are specifically tailored to a specific subset of the training proteins. In iterations 3–4, we note that 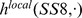 occurs slightly more often than the other feature functions (6 times over 20). From the two last rows, which give the averaged cross-validated scores and the averaged verification scores, we observe that while the cross-validated score systematically increases, the verification score becomes unstable after the two first iterations. These observations reinforce the fact that the selected feature functions become more and more specific to training samples. From these results, it is clear that the feature functions 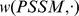 and 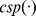 bring the major part of the predictive power that can be obtained by our feature functions.

According to these results, we focus in the following on the feature functions 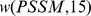, 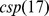 and 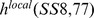, where we chose windows sizes by taking the average sizes reported in Table 4. Note that, contrarily to the observation of Figure 4 that suggested large feature function sets, our method carefully selected a very small number of relevant feature functions that led to a more simpler and still very accurate classifier.

### Evaluation of the constructed prediction pipeline

We now compare our constructed prediction pipeline with the state of the art. We consider three baselines that were evaluated using the same experimental protocol as ours (10 cross-validated 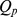). The first baseline is the recursive neural network approach proposed by Cheng *et al.* [6]. These authors, who introduced the SPX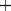 dataset, reached a pattern accuracy of 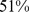 using the true secondary structure and solvent accessibility information. Lin *et al.* [7] proposed to predict the bonding state probabilities using a fine tuned support vector machine. They obtained a pattern accuracy of 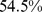 by using the same data for feature selection and for evaluation, making this results probably over-estimated. Vincent *et al.* [47] proposed a simple approach based on a multiclass one-nearest neighbor algorithm that relies on the fact that two proteins tend to have the same disulfide connectivity pattern if they share a similar cysteine environment. This method reaches a pattern accuracy of 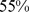.

Table 5 reports the performance obtained by ETs with feature functions 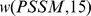, 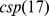 and 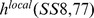. We observe that using only 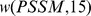 already leads to a pattern accuracy of 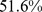, which is better than the baseline of Cheng *et al.* [6]. A significant improvement of 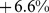 is achieved by adding the feature function 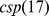, which leads to a model that significantly outperforms the state of the art. The feature function 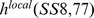 leads to small further improvement of the 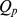 score, but due to the large variance, this improvement cannot be shown to be significant.

**Table 5:** Evaluation of the proposed prediction pipeline.

Features	Q*_p_*
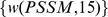	51.6% 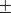 3.58%
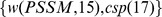	58.2% 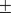 2.74%
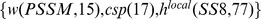	58.3% 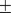 3.04%
**Baseline**	
Vincent *et al.* [47]	55.0%
Lin *et al.* [7]	54.5%
Cheng *et al.* [6]	51.0%

We report the mean and standard deviation of the 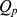 scores obtained using 10-fold cross-validation on the SPX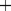 dataset.

From these results, we conclude that only the following two feature functions are sufficient for high-quality disulfide pattern prediction in combination with ETs: local PSSM windows and CSP windows. Note that it might be the case that, by using larger datasets, feature functions such as medium-size histograms on predicted DSSP secondary structure could slightly improve the quality of the system.

Table 6 reports the pattern accuracy as a function of the true number of disulfide bridges. By comparing the results with the three baselines, we observe that our method outperforms the baselines, except for proteins with 4 potential disulfide bonds where the approach proposed by Vincent *et al.* [47] obtains a better pattern accuracy.

**Table 6:** Comparison of 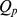 scores on SPX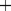.

Number of bridges	Cheng *et al.*	Vincent *et al.*	Lin *et al.*	Becker *et al.*
	(2006)	(2008)	(2009)	(2013)
1	59%	59%	60.6%	61.8%
2	59%	63%	65.9%	66.6%
3	55%	64%	59.8%	67.6%
4	34%	48%	36.4%	41.8%
all	51%	55%	54.5%	58.3%

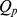
 scores obtained using 10-fold cross-validation.

### Sensitivity of extremely randomized trees to its hyper-parameters

This series of experiments aims at studying the impact of the hyper-parameters (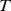, 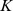 and 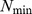) when using the feature functions 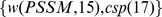. With these two feature functions, the number of features is 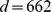. The default setting is 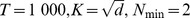 and we study the parameters one by one, by varying their values in ranges 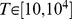, 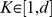 and 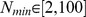.

**Figure 6:**
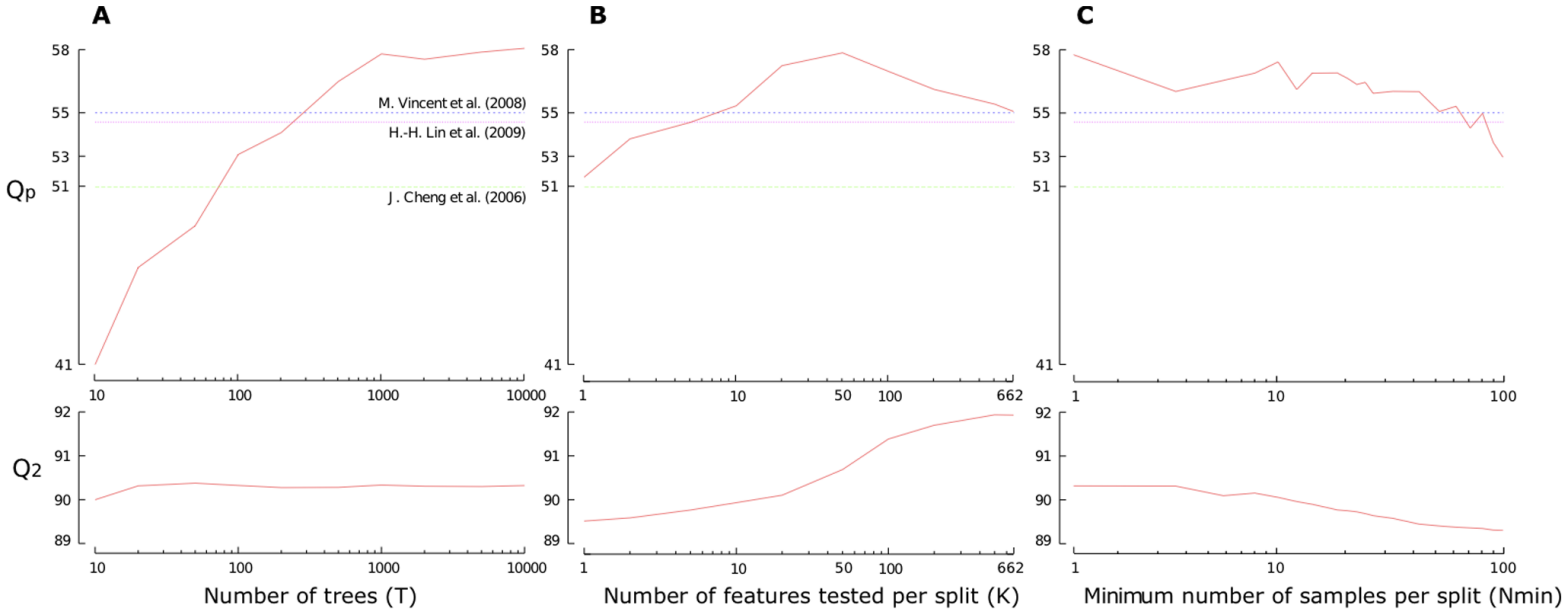
Sensitivity of ETs w.r.t. hyper-parameters. The experiments are performed on the SPX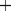 dataset. We used the two feature functions 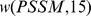 and 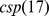. (A) Impact of the number of trees 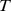 (from 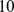 to 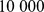) with 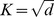 and 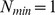, where 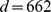 is the number of features. (B) Impact of 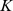 (from 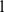 to 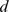) with 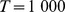 and 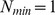. (C) Impact of 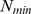 (from 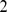 to 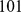).

Figure 6 reports the 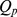 and 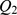 scores in function of the three hyper-parameters. As a matter of comparison, we also reported the 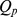 scores of the three baseline described previously. We observe that the 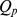 score grows (roughly) following a logarithmic law w.r.t. 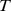. The value of 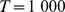 occurs to be very good tradeoff between performance and model complexity. Concerning 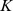, we observe that the value maximizing 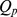 is 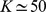, which is a bit larger than the default setting 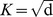. Note that the protein-level performance measure 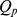 and the cysteine-pair level performance measure 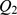 do not correlate well in terms of the effect of parameter 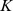, which confirms the interest of directly optimizing 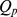 in our feature function selection algorithm. 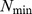 controls the complexity of built trees and, hence, the bias-variance tradeoff by averaging output noise. It is usually expected that a small value of 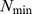 improves performance. In our case, we observe that increasing 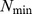 never improves the performance measures and that 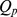 has a large variance.

## Results: Chain Classification and Cysteine Bonding State Prediction

Until now, our pipeline relies on a perfect graph matching algorithm that always attempts to predict patterns involving all cysteines. Due to this, our approach is, for the moment, unable to deal with partially connected disulfide patterns (except for proteins with an odd number of cysteines having a single non-bonded cysteine). This can be harmful, especially on datasets containing many non-bonded cysteines. For example, if we apply our pipeline to the SPX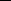 dataset, the pattern accuracy 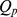 is only 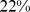, since most proteins of this dataset do not contain any disulfide bridges. We now focus on this issue by coupling our predictor with filters based on the output of a chain classifier and on the output of a cysteine bonding state predictor. We first construct a chain classifier and a cysteine bonding state predictor by applying our feature function selection approach. We then study combinations of these predictors with our disulfide pattern predictor.

### Chain classification

We consider the *binary* chain classification problem, which consists in classifying proteins into those that have at least one disulfide bridge and those that have no disulfide bridge. In order to construct a chain classifier, we apply the same methodology as before: we perform feature function selection on top of extremely randomized trees. Since chain classification works at the level of proteins, the set of candidate feature functions is restricted to labels global histograms. We also include as candidates the simple feature functions returning the number of residues, the number of cysteines and the parity of the number of cysteines. We use the following default setting for ETs: 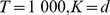 and 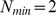. According to preliminary experiments, we found out 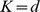 to be a good default setting for this task. This is probably due to the fact that we have far less features than we had before.

We performed ten runs of the feature function selection algorithm on the SPX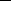 dataset, which contains both proteins without disulfide bridge and proteins with disulfide bridges. The performance measure is the accuracy, i.e., the percentage of proteins that are correctly classified. In every feature function selection run, the first selected feature function was 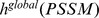 and the second one was 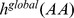. Starting from the third iteration, the results are more diverse and the system starts to overfit. By keeping the two first feature functions, we reach a 10 fold cross-validation accuracy of 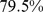 on SPX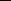, which is not very far from the 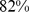 accuracy obtained by [47].

### Cysteine bonding state prediction

Cysteine bonding state prediction consists in classifying cysteines into those that are involved in a disulfide bridge and those that are not. To address this task, we apply our feature function selection approach on top of extremely randomized trees (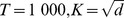 and 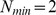). The set of candidate feature functions is composed of those depending only on the protein (number of residues, number of cysteines, parity of the number of cysteines, labels global histograms) and those depending on the protein and on a single cysteine (labels local histograms, labels local windows, cysteine separation profile window). We consider the same window size values as in previous section. The evaluation measure is binary accuracy, i.e., the percentage of cysteines that are correctly classified.

We ran the feature selection algorithm once for each of the ten different train/test splits of SPX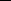. We observed that the selected feature functions set 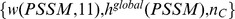 led to a binary accuracy of 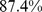, which outperforms the result of 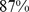 obtained by Vincent *et al.* [47]. On SPX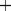, we obtain a similar accuracy of 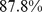.

Note that once we have a cysteine bonding state predictor, we can use it to also solve the chain classification task as follows. In order to predict whether a protein contains disulfide bridges or not, we run the cysteine bonding state predictor on each cysteine, and see if at least one cysteine is predicted as being bonded. By applying this strategy to SPX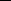, we obtain a chain classification accuracy of 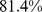, which is comparable to the score of [47].

Table 7 summarizes the feature functions that were selected for the three tasks that we consider in this paper.

**Table 7:** Selected feature functions of the three tasks.

Task	Features
Chain classification	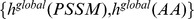
Cysteine bonding state prediction	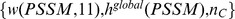
Disulfide pattern prediction	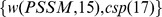

The feature functions were determined by the application of our selection algorithm on top of extremely randomized trees, using the SPX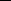 dataset for chain classification and cysteine bonding state prediction and the SPX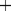 dataset for disulfide pattern prediction.

### Impact on pattern prediction

Now that we have constructed a chain classifier and a disulfide bonding state predictor, we focus on the question of how to exploit the corresponding predictions in order to improve disulfide pattern prediction. Note that, in some cases, the user may have prior knowledge of either the chain class (whether the proteins contains any disulfide bridges or not) or of the cysteine bonding states (which are the cysteines that participate to disulfide bridges). To take the different possible scenarios into account, we study the following four settings:

- *Chain class known:* in this setting, we assume that the chain classes are known a priori and simply filter out all proteins that are known to not contain any disulfide bridge. For the proteins that contain disulfide bridges, we run our disulfide pattern predictor as in previous section.
- *Chain class predicted:* in this setting, we replace the knowledge of the chain class by a prediction. We therefore rely on the chain classifier derived from the cysteine bonding state predictor, which obtained a chain classification accuracy of 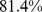.
- *Cysteine states known:* we here assume that the bonding states of cysteines is known a priori. We modify the disulfide pattern predictor by setting a probability of zero to any cysteine pair containing at least one non-bonded cysteine.
- *Cysteine states predicted:* in this setting, we first run our cysteine state predictor and then perform disulfide pattern prediction by only considering cysteine pairs in which both cysteines where predicted as bonded.

Note that, since the SPX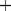 dataset is entirely composed of proteins with at least one bridge, our two first settings based on chain classification are irrelevant for this dataset. In these experiments, we learnt models using a 10-fold cross-validation of ETs (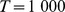,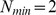 and 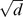).

Table 8 summarizes the results of our experiments on chain classification, cysteine bonding state prediction and disulfide pattern prediction with our four different settings. When the chain classes are known, we observe a significant improvement of the 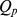 score: from 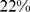 to 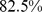 on SPX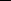. When replacing the true chain classes with predicted chain classes, we still have a relatively high 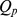 score: 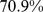. This result is detailed in Table 9 as a function of the true number of disulfide bridges. We observe that our method clearly outperforms the method of Vincent *et al.* [47] on proteins containing one or two disulfide bonds and performs slightly worst on proteins with three disulfide bonds. Given that a majority of proteins in SPX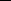 contain less than two bonds, these results leads to an overall score that is significantly better than that of Vincent *et al.* When the cysteine bonding states are known, we obtain impressive disulfide pattern accuracies: more than 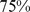 on SPX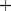 and almost 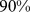 on SPX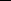. When using predicted cysteine bonding states, we still observe an impressive improvement on SPX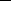: from 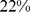 to 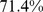. However, on SPX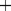, the score slightly degrades (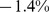). This is probably related to the fact that, as soon as one cysteine is falsely predicted as being non-bonded, it becomes impossible to recover the correct disulfide pattern.

**Table 8:** Evaluation of the three tasks.

Filter	SPX 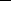	SPX 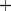
**Chain classification**		
–	79.5% 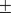 2.40%	–
Cysteine states predicted	81.4% 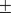 2.66%	–
**Cysteine bonding state prediction**		
–	87.4% 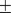 1.14%	87.8% 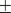 2.20%
**Disulfide pattern prediction**		( 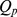 )
–	22.0% 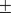 2.00%	58.2% 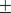 2.74%
Chain class known	82.5% 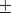 2.24%	–
Chain class predicted	70.9% 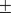 3.10%	–
Cysteine states known	89.9% 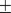 1.57%	75.8% 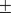 2.09%
Cysteine states predicted	71.4% 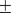 2.76%	56.8% 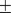 2.52%

We report the mean and standard deviation of the binary accuracy for chain classification and cysteine bonding state prediction while the 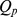 score is used for disulfide pattern prediction. The symbol – indicates that all cysteines are used in the experiment.

**Table 9:** Comparison of 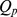 scores on SPX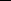 when chain classes are predicted.

Number of bridges	Vincent *et al.*	Becker *et al.*
	(2008)	(2013)
1	30%	77.1%
2	49%	63.5%
3	61%	60.4%
4	37%	44.2%
all	39%	70.9%

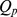
 scores obtained using 10-fold cross-validation.

## Discussion

Disulfide connectivity pattern prediction is a problem of major importance in bioinformatics. Recent state of the art disulfide pattern predictors rely on a three step pipeline, in which the central component is a binary classifier that predicts bridge bonding probabilities given cysteine pair representations. However, the comparison of the conclusions of these works is difficult because it is often the case that these different studies rely on different kinds of binary classifiers and slightly differ in their experimental protocol. Therefore, the relevance of some features is still a subject under heavy debate. This paper has proposed an extensive study on the best way to represent cysteine pairs in the form of features. We considered three classification algorithms: k-nearest neighbors, support vector machines and extremely randomized trees, and we proposed a forward feature function selection algorithm that we applied on the standard benchmark dataset SPX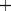.

Our experiments have shown that extremely randomized trees (ETs) are highly promising in terms of predicted disulfide pattern accuracy 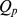. ETs are easy to tune and thanks to their use of decision trees, they benefit from good scaling properties, making them applicable to large sets of training proteins and large sets of features. The result of our feature selection experiments with ETs is that the primary structure related features functions 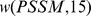 (a local window of size 15 on the evolutionary information) and 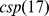 (a window of size 17 on the cysteine separation profile) are sufficient to reach a very high performing disulfide pattern predictor: ETs with these two kinds of features predict correct disulfide connectivity patterns in 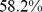 of proteins, which outperforms the state of the art [47] with a 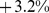 improvement. Furthermore, we showed that appending any other feature function does not lead to significant subsequent improvements or even decreases the accuracy.

We also investigated the question of how to exploit our disulfide pattern predictor with filters based on the output of either a chain classifier or of a cysteine bonding state predictor. Among the four scenarios that we considered, we observed an important potential for improvement when the cysteine bonding states are known, with scores reaching 75% on SPX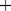 and almost 90% on SPX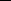. When using predicted cysteine bonding states, we still observe an impressive improvement on SPX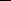 (from 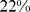 to 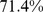) but the score slightly degrades (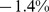) on SPX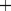. This degradation is probably due to the fact that, as soon as one cysteine is falsely predicted as being non-bonded, it becomes impossible to construct the correct disulfide pattern. Therefore, one direction of future research is to develop more sophisticated methods to couple the cysteine bonding state prediction task with the pattern prediction task. One direction for such a better coupling is to apply the ideas developed in [32] on multi-stage and multi-task prediction, *e.g.*, by iteratively re-estimating the disulfide bond probabilities.

Note that despite the fact that several studies have shown that tree-based ensemble methods often reach state of the art results in supervised learning (see e.g. [43]), these methods were surprisingly few applied to structural bioinformatics problems yet. We believe that ETs in combination with feature function selection provide a general methodology that can be applied to a wide range of protein related prediction problems and more generally to any kind of classification problems involving many different possible representations.
